# Implementing Evidence-Based Alcohol Interventions in a Resource-Limited Setting: Novel Delivery Strategies in Tomsk, Russia

**DOI:** 10.3109/10673229.2012.649121

**Published:** 2012-02-15

**Authors:** Sonya S Shin, Viktoriya Livchits, Adrianne K Nelson, Charmaine S Lastimoso, Galina V Yanova, Sergey A Yanov, Sergey P Mishustin, Hilary S Connery, Shelly F Greenfield

**Affiliations:** 1 Harvard Medical School, Brigharn & Women's Hospital, Boston, MA; 2 Division of Global Health Equity, Brigharn & Women's Hospital, Boston, MA; 3 Partners in Health—Russia, Boston, MA; 4 Tomsk Oblast Clinical Tuberculosis Hospital, Belmont, MA; 5 Tomsk Oblast Tuberculosis Dispensary, Belmont, MA; 6 Tomsk, Russian Federation; McLean Hospital, Belmont, MA

**Keywords:** alcohol, global health, implementation, resource-limited settings, resource-poor settings, Russia, tuberculosis

## Abstract

Effective implementation of evidence-based interventions in “real-world” settings can be challenging. Interventions based on externally valid trial findings can be even more difficult to apply in resource-limited settings, given marked differences—in provider experience, patient population, and health systems—between those settings and the typical clinical trial environment. Under the auspices of the Integrated Management of Physician-Delivered Alcohol Care for Tuberculosis Patients (IMPACT) study, a randomized, controlled effectiveness trial, and as an integrated component of tuberculosis treatment in Tomsk, Russia, we adapted two proven alcohol interventions to the delivery of care to 200 patients with alcohol use disorders. Tuberculosis providers performed screening for alcohol use disorders and also delivered naltrexone (with medical management) or a brief counseling intervention either independently or in combination as a seamless part of routine care. We report the innovations and challenges to intervention design, training, and delivery of both pharmacologic and behavioral alcohol interventions within programmatic tuberculosis treatment services. We also discuss the implications of these lessons learned within the context of meeting the challenge of providing evidence-based care in resource-limited settings. (Harv Rev Psychiatry 2012;20:58–67.)

## INTRODUCTION

Alcohol use disorders (AUDs) are undertreated in the United States and worldwide, despite their high medical costs, social consequences, and role in the incidence and severity of other diseases—in particular, diseases of poverty. In the United States, AUDs rank seventh among the leading causes of premature death and disability, as measured in disability-adjusted life years.^1^ In addition to contributing heavily to chronic morbidity, AUDs contribute causally to 13 of the top 20 causes of mortality.^1^,^2^ Yet, such figures in the United States pale in comparison to global estimates of the burden of AUDs. The countries with the highest alcohol consumption levels in the world are clustered in eastern Europe, particularly in Russia and the other countries of the former Soviet Union, though lower-income countries in southern Africa and South America also have exceptionally high rates of consumption.^2^ Alcohol dependence is an established cause of 12 of the 20 leading global causes of death.^2^,^3^ It is therefore not surprising that alcohol-attributable deaths have risen in the past decade.^2^ Worldwide, the World Health Organization (WHO) estimates alcohol use caused 3.2% of deaths and 4.0% of total disability-adjusted life years in 2009.^4^ Despite growing recognition of the global impact of AUDs, particularly in lower- and middle-income countries,^4^ the health services available for diagnosing and treating AUDs in such settings are appallingly scarce.^5^,^6^ In addition, despite the clear evidence that screening and counseling for AUDs is highly cost-effective and likely cost saving,^7^ evidence-based screening tools and interventions are often difficult to implement in resource-limited settings because of marked differences in provider experience, patient populations, and health systems.

Russia provides an instructive example of the disparity between the overwhelming need for alcohol care and the limited options available to most individuals, particularly those marginalized by poverty and stigma. Alcohol consumption has contributed to the marked rise in mortality in Russia over the past two decades. Between 1992 and 2001, an excess of 2.5–3 million deaths among Russian adults in middle age occurred over a projection based on 1991 mortality estimates.^8^ Most experts consider alcohol to be the most important factor driving these trends, implicated directly or indirectly in approximately one-third of all Russian deaths.^9^–^13^ Although little data are available for alcohol use in Tomsk, the available information suggests that it falls toward neither the high nor low extreme for Russian oblasts.^14^ One study led by the Russian Cancer Center gathered mortality data from 50,066 households in Tomsk, Bernaul, and Biysk with a deceased member from 1990 to 2001. They found that 52% of total mortality for adults 15–54 years could be attributed to alcohol use and that the changes in the number of alcohol-related deaths may account for the large fluctuation of mortality in Russia as a whole during that period.^15^ Another study using anatomical pathology to examine causes of violent death in the Tomsk area observed the presence of ethyl spirit in 63.2% of bodies examined.^16^

The convergence of AUDs with other diseases of poverty has implications for Russia's ability to control the rising tides of tuberculosis (TB) and human immunodeficiency virus (HIV) disease.^17^,^18^ TB patients with AUDs are at increased risk of poor treatment outcomes such as default, failure, and death.^19^ In a recent study, it was determined that 55% of TB patients in St. Petersburg and 70% in Ivanovo had AUDs.^20^ Also intimately linked to alcohol use is the HIV epidemic in Russia, which affects a larger percentage of its inhabitants than any other European country. In Russia, alcohol use is consistently and strongly associated with HIV risk behavior across a spectrum of high- and low-risk populations.^21^–^28^ Abdala and colleagues^23^ found that in St. Petersburg both intravenous drug users and those not using IV drugs were more likely to have multiple sexual partners if they reported having sex while drinking alcohol. Alcohol use has also been linked to increased drug-related risk in St. Petersburg, including use of unclean needles.^24^ The impact of alcohol use on TB and HIV risk in Russia reflects associations that have been well documented at the global level.^2^,^29^–^33^

Underdiagnosis, undertreatment, and lack of evidence-based interventions (EBIs) are the norm for alcohol management in most of Russia.^20^,^34^ Because AUDs are generally managed by addiction specialists (narcologists), general practitioners rarely either screen for AUDs or offer brief counseling interventions (BCIs).^13^ Patients have numerous disincentives for seeking alcohol care, including out-of-pocket payment for services and their official registry within Narcology Services, which can result in difficulty seeking employment, employment loss, and restrictions in owning or driving a car. Medical culture in Russia is heavily reliant on pharmacologic therapies, but the Russian health care system provides limited access to evidence-based pharmacotherapies for AUDs—which are often prohibitively expensive anyway.

In many resource-limited settings, structural factors (in addition to individual-level characteristics) strongly shape health-seeking behavior and one's ability to adhere to treatment.^35^ In order to overcome such barriers to alcohol care, innovative health delivery strategies must integrate routine diagnosis and treatment into services that are free of charge and readily accessed by marginalized patients who would not otherwise seek alcohol treatment. One example of strong infrastructure for such settings is WHO's suggested strategy for managing TB: Directly Observed Treatment, Short-Course, or DOTS.^36^ This therapy is considered the international standard for TB management in many lower- and middle- income countries, and includes directly observed treatment with TB medications and programmatic components (e.g., outreach to find individuals who default from care, and incentives and enablers such as transportation and meal vouchers to help patients attend their daily visits) to keep individuals in TB care for the six months required to complete treatment (i.e., case holding). In essence, DOTS is designed with programmatic elements to overcome many of barriers to health services experienced by marginalized patients in resource-limited settings.

To take advantage of the existing DOTS infrastructure, we developed an integrated model of alcohol and TB care that would deliver screening and treatment services for individuals with AUDs as part of routine TB services. We then implemented these strategies (under both programmatic and research auspices) within the Tomsk Oblast Tuberculosis Services, in an effort to decrease alcohol-related morbidity and improve TB treatment outcomes. This article provides a case study of how these interventions were conceptualized, developed, and implemented, and highlights lessons learned that could inform the replication of such programmatic interventions in other parts of Russia and elsewhere.

## METHODS

### Study Setting

Our interventions took place in the Tomsk Oblast of western Siberia, Russia, an area of 316,900 km^2^, with a population of 1,200,000. As previously described, Russia has one of the highest rates of alcohol consumption per capita in the world.

In 2010, TB incidence in Tomsk was 73.3 per 100,000, and TB mortality was 8.6 per 100,000.^37^ TB treatment services in Tomsk City are provided in three facilities (inpatient hospital, polyclinic, and day hospital). In 1991, the Tomsk Oblast Tuberculosis Services implemented WHO standards for TB care (i.e., DOTS).^36^ In 2000, it began a collaborative project with our nongovernmental organization, Partners in Health, to improve TB treatment outcomes. Patients receive TB therapy daily under direct observation, which is provided free-of-charge for a minimum of six months. Prior to the efforts described below, AUD management was not integrated into TB services. Physicians generally referred patients to narcologists, who were solely responsible for AUD diagnosis and management. Free on-site addiction care was available for hospitalized patients who agreed to see the narcologist. Treatment options included detoxification for alcohol intoxication, disulfiram, and psychotherapy; however, BCIs and naltrexone (NTX) were not available.

Through a collaborative effort that began in 2004 and involved Brigham and Women's Hospital, McLean Hospital, Partners in Health, and the Tomsk Oblast Tuberculosis Services, we established the Tomsk Tuberculosis Alcohol Working Group (Working Group) comprising U.S. and Russian experts to develop and implement programmatic and research efforts to address AUDs among TB patients in Tomsk. Strategies were implemented in two areas: (1) screening and specialist referral; and (2) integrated alcohol treatment. Screening and referral strategies using the Alcohol Use Disorder Identification Test^38^ (AUDIT) were implemented as programmatic activities with support from the Global Fund to Fight AIDS, TB and Malaria, and have been described elsewhere.^39^ Treatment interventions were implemented under the auspices of research funded by the United States' National Institute of Alcohol Abuse and Alcoholism.

**Figure 1:**
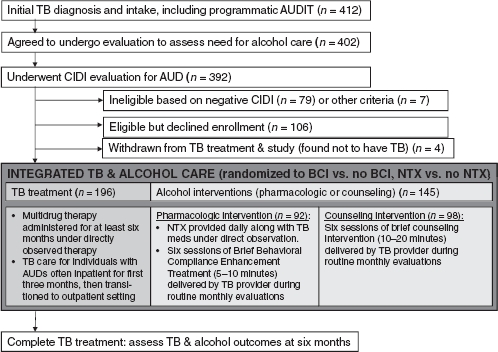
Flow chart of therapeutic intervention. AUD, Alcohol use disorder; AUDIT, Alcohol Use Disorder Identification Test; BCI, brief counseling intervention; CIDI, Composite International Diagnostic Interview; NTX, naltrexone; TB, tuberculosis.

This study, Integrated Management of Physician-Delivered Alcohol Care for Tuberculosis Patients (IMPACT), was a randomized, controlled trial to assess the effectiveness of NTX or monthly BCIs for TB patients with AUDs as an integral part of routine care at the Tomsk Oblast Tuberculosis Services in Tomsk, Russian Federation.^40^ The study took place in both inpatient and ambulatory sites of TB services in Tomsk City. As is typical for TB treatment, patients were initially enrolled in the hospital and then transitioned to ambulatory care once medically stable. A total of 412 individuals were recruited, and 200 of the 306 eligible individuals were enrolled. We assessed alcohol and TB treatment outcomes at six months to determine the effect of both interventions on change in alcohol consumption and favorable TB treatment response. A total of seven physicians delivered study interventions. Of note, this was an effectiveness trial intended to assess the impact of evidence-based alcohol interventions when integrated into Russian TB health services. We therefore designed study interventions with the intent of integrating alcohol care seamlessly into routine TB care. See Figure 1.

### Data Collection

We collected qualitative data using multiple methods to allow for triangulation and verification of our findings. Investigators trained in ethnography carried out participant observation during the six years of the project, including two years of preparation with Tomsk collaborators and four years of trial execution. For qualitative evaluation, we defined participants as TB patients with AUDs who enrolled in the study (study subjects), eligible patients who were recruited but declined enrollment, and TB providers who delivered study interventions and collected study data. Field notes from observation of participants were analyzed for theme and content, and findings were used to develop semistructured interview guides for one-on-one interviews and focus group discussion. Four one-on-one interviews and five focus group discussions consisting of semistructured, open-ended questions were conducted between 2008 and 2011. The objectives of these interviews were to understand participant attitudes toward the alcohol interventions that were implemented. Questions elicited opinions regarding the appropriateness of the study interventions (BCI and NTX), their effectiveness, problems or limitations in application, and relevance for programmatic use. Individual interviews were conducted with three of the TB physicians. We conducted focus groups with participating providers at two separate times; in addition, we conducted three focus groups involving the following patients: (1) individuals who had declined enrollment; (2) subjects randomized to BCI; and (3) subjects randomized to NTX. Patient participants were selected by asking the study team to identify four to six candidates for each group. We invited candidates beforehand and obtained oral consent for participation using a written script for all who attended. All but one individual agreed to participate in focus group discussions. A study staff trained in conducting focus group discussions led the interviews, with an observer present. Both the facilitator and observer took notes. Sessions lasted approximately one hour and were tape-recorded. An individual fluent in Russian reviewed recordings and coded them for theme and content. We also asked subjects who had finished the assigned treatment to complete written exit surveys six months later, at the final alcohol-assessment interview. Surveys asked subjects about their experience in the trial, whether they felt any improvement in their AUDs, and whether they perceived their assigned study interventions to be effective. We organized data into four areas: intervention design, training, intervention delivery, and outstanding challenges.

### Ethical Considerations

The Partners Human Subjects Committee at Brigham and Women's Hospital and the Ethics Committee of State Research Center of Virology and Biotechnology “Vector” in Novosibirsk, Russia, both approved the clinical trial.

## FINDINGS

### Intervention Selection

Initial efforts of the Working Group focused on identifying EBIs that could be readily applied as part of TB care. We considered both behavioral and pharmacological interventions, and weighed the pros and cons of both. Tomsk colleagues felt that behavioral interventions would potentially be more acceptable to some patients who would not want to take more medications, though some reservations were voiced regarding the appropriateness and feasibility of providers not trained in addictions care to deliver counseling on AUDs. Another factor was that, since prevailing Russian medical culture relies heavily on pharmacologic therapy, both providers and patients might place more “faith” in, and therefore favor, pharmacologic over behavioral interventions. Based on our literature review, we considered two interventions—BCI and NTX—to be acceptable for use among TB patients. Literature review and expert opinion provided reassuring experience that BCI could be effectively delivered by nonspecialists and that no pharmacologic interactions occurred between NTX and anti-TB medications. We further modified our pharmacologic intervention in response to new data that medical management combined with NTX was more effective than NTX alone.^41^

### Intervention Adaptation

Once the Working Group identified the most appropriate interventions, we adapted these interventions to the local context, taking into consideration the providers, patients, and health system. We identified existing behavioral interventions. For BCI, we used the National Institute on Alcohol Abuse and Alcoholism's 2005 manual: *Helping Patients Who Drink Too Much: A Clinician's Guide*. For medical management, we sought an intervention that would focus on medication adherence and on goal setting to reduce alcohol consumption, but that maintained a clear distinction from BCI by avoiding both counseling that used problem-solving methods and the style and techniques of motivational interviewing. We identified the Brief Behavioral Compliance Enhancement Treatment (BBCET) that was developed for the Topiramate Clinical Protocol,^42^ and obtained permission from Johnson and colleagues to adapt the manual.

While retaining the core components of each behavioral intervention, we modified areas of content, style, and format to be culturally and medically appropriate for our target population. Our team developed adapted manuals and translated them into Russian. In terms of content, we adapted the BCI to highlight the relevance of AUDs for TB patients; we therefore provided feedback and education on the effect of AUDs on TB risk, on the potentially increased side effects from TB therapy, and on alcohol's detrimental effect on treatment outcomes. The BBCET manual included guidelines for use of NTX in TB patients, including management of side effects potentially caused by both NTX and TB medications, perioperative management of NTX to avoid adverse reactions due to anesthesia and pain control, and a description of contraindications for NTX use, such as pregnancy and opiate use. Stylistic modifications resulted from careful consideration of how to deliver the BCI using motivational interviewing in a way that would be natural in the context of typical Russian patient-provider interactions (traditionally a more authoritative relationship than in the United States). For instance, multiple bilingual collaborators carefully reviewed the wording chosen for the manuals in order to ensure that the language was neither too medical nor too informal. This aspect of adaptation required input by Russian providers and Russian-speaking individuals trained in motivational interviewing because the wording had to be empathic yet respect the traditional lines of authority in the patient-provider relationship.

As an example of cultural challenges, many TB patients have been previously incarcerated and have been inculcated in a prison culture with its rules and mentality, including mistrust of authorities. Many TB doctors have worked with TB patients for so long that they accept these social norms, rather than try to gain the trust of the patient or provide empathic support.

By introducing motivational-interviewing techniques, we confronted and, in some cases, overcame these beliefs and behaviors on both sides of the patient-physician relationship. BCI encourages both patients and providers to take an active role in exploring changes in drinking behavior. We found that physicians and patients were more comfortable when the BCI dialogues initially covered neutral topics and then slowly moved into drinking-related problems and patients' feelings toward their drinking. Over time and with continuous coaching, TB providers learned to be empathic and provide encouragement; they were unexpectedly rewarded by patients' positive responses to this type of behavior. Our team also modified the format of both interventions to be folded into routine TB encounters. The BCI incorporated routine aspects of the medical encounter, such as assessment of laboratory analysis and clinical response, as a way of providing information and feedback to the patient using motivational interviewing techniques. Likewise, to integrate treatment delivery, health care workers administered NTX along with TB medications under directly observed treatment and recorded adherence to NTX using a form similar to that for monitoring TB treatment.

### Initial Training and Ongoing Supervision

We used a train-the-trainer model for initial and ongoing training of Tomsk TB physicians. U.S. addiction experts trained two providers, including a Russian physician who was based in Tomsk for the duration of the study. Training of TB physicians took place prior to initiating the study and included didactic teaching that reviewed both interventions, including the theoretical framework, literature establishing efficacy, and Tomsk-specific adaptations. In addition, trainees watched a video of mock interviews (one using motivational-interviewing style, and one not) and participated in interactive role-play to practice motivational-interviewing techniques. Most of the providers were not familiar with role playing, and not all individuals were willing to practice role playing. Provider competency was required in order to participate in the study. We assessed competency by performing mock sessions (BCI and BBCET) with the Tomsk-based trainer. We also modified our training to include a one-on-one “practice session” just prior to competency assessment, in which the Tomsk-based trainer role-played the patient. This addition allowed for nonthreatening, individualized feedback and improved the quality of the competency-assessment interviews. All providers who participated in competency assessment were certified.

As part of the research protocol, we established fidelity procedures that not only served to assess adherence to the behavioral study intervention but also provided ongoing supervision and improved provider proficiency during the course of the study. The fidelity-assessment team included the Tomsk-based trainer, an addiction specialist with clinical and research experience, and two Russian-speaking staff based in the United States who were responsible for reviewing audiotapes of study encounters and rating providers using a standardized fidelity-assessment form that included constructive feedback. We requested that all study encounters be audiotaped (if subjects allowed), and approximately 10% of the encounters were reviewed by the fidelity team. Weekly teleconferences enabled the U.S.-based team to receive feedback from the Tomsk trainer regarding the clinical realities and challenges occurring in the field and also enabled the trainer to better understand the fidelity reviewers' reports and to relay constructive feedback to the providers. Related to the pharmacologic intervention, we conducted regular teleconferences in the first year of the study with U.S.-based specialists to review “difficult cases” of individuals receiving NTX. Discussion included management of surgical cases, NTX-related side effects, and interruption of NTX due to binges and detoxification. We modeled case conferences after case-discussion meetings held with Tomsk providers when treatment of multidrug-resistant TB was being introduced into the Tomsk TB program. This ongoing accompaniment—that is, continual coaching and feedback to providers during the “learning curve” of implementation—represents a labor-intensive necessity for behavioral research but is an aspect of ongoing supervision that is often overlooked during programmatic implementation. In our experience, however, such accompaniment is crucial in obtaining local “buy-in” and establishing true clinical proficiency among participating providers.

### Intervention Delivery

At the outset we developed study protocols describing the delivery of both interventions. Once “in the field,” however, many details of intervention delivery presented challenges requiring further adaptation to the setting. Responding to the needs of all participants (program directors, providers, patients), we made several decisions in the course of the project that, rather than involving amendments to the study protocol, constituted logistical refinements that allowed us to successfully implement the study protocol. Prior to beginning IMPACT, we had already implemented the written AUDIT as part of the initial evaluation for all patients starting TB treatment. This screening instrument allowed us to identify individuals at risk for AUDs and to refer them for more detailed assessment. Having such a streamlined mechanism in place prior to study start was helpful in two ways. AUDIT training increased TB providers' general awareness of AUDs, and the providers themselves quickly identified high-risk patients and referred them to the study. Based on our validation study, an AUDIT score of ≥8 had a sensitivity of 83.5% in this population, compared to a diagnosis of alcohol abuse or dependence based on the Composite International Diagnostic Interview (CIDI).^43^

During the course of the study, we eventually opted to screen all individuals with the CIDI in order to maximize study enrollment. Consistent with our validation data, we identified 51 patients (12.8% of the total cohort) who scored less than 8 on the written AUDIT questionnaire but were diagnosed with AUDs when interviewed using the more detailed, verbally administered CIDI interview. Often, their providers suspected AUDs in such individuals despite AUDIT results. The programmatic implications of this experience are that AUDIT serves as a useful screening tool but that a more careful, personalized assessment should be pursued in cases suspected of having AUDs despite negative AUDIT screens.

Although NTX is approved for the treatment of AUDs in Russia, access to this medication is limited, and it is used mainly to treat private patients for heroin addiction.^44^ Moreover, the Russian pharmaceutical market was unstable during our study, with frequent regulation and price changes. We maintained a surplus supply of NTX, which ensured continuous treatment despite disruptions in purchasing mechanisms from contracted vendors. Several administrative steps were also taken to integrate NTX into TB care: obtaining authorization from the TB program to use NTX; and training ancillary staff to administer NTX along with TB medications under direct observation, to monitor for side effects, and to record administration.

Despite initial concerns that patients would reject NTX due to the increased pill burden, this occurred with only three participants. In fact, all providers and patients who participated in focus group discussions and 75% of patients who completed the exit survey perceived the medication to be effective. Overall, the mean adherence to NTX was 81.5% (standard deviation = 37.4%), defined as the percentage of doses ingested in relation to the total number of doses prescribed prior to study completion or withdrawal. For reference, the mean adherence to DOTS for TB therapy was 86.7% (standard deviation = 8.5%). Such high rates of NTX adherence likely reflect the delivery system designed to streamline administration and present minimal additional burden to staff and patients. NTX adherence was higher in the first three months of therapy than in the last three months (85% vs. 74%). This trend could be due to greater logistical challenges in administering directly observed NTX when patients transitioned to outpatient care, as they increased their binge drinking (*zapoi*) and tended to miss daily appointments.

In terms of delivering the behavioral interventions, we envisioned both the BCI and BBCET to be incorporated into routine monthly TB evaluations delivered by TB physicians. Although both interventions were brief (15–20 minutes for BCI; 5–10 minutes for BBCET), their delivery did involve an additional time burden during routine clinical encounters. Importantly, the TB hospital director actively supported this collaboration and allowed providers to have protected time to carry out study protocols. We did not provide patient incentives for counseling sessions (only for data-collection interviews) in order to avoid introducing an artificial incentive for attending BCI and BBCET sessions.

Manuals for both BCI and BBCET provided structure that helped providers deliver the interventions correctly—particularly in terms of content and format. Adherence to BCI style (motivational interviewing), however, was more challenging. Becoming proficient and comfortable in motivational interviewing required practice and ongoing feedback, as previously described. Serial focus group interviews with providers revealed that initial skepticism toward the behavioral interventions eventually gave way to endorsement and perceived effectiveness. Overall, among the scheduled BCI and BBCET encounters, 51% of BCI and 57% of BBCET encounters were successfully delivered. Similar to trends observed with NTX, a greater proportion of sessions was completed during the first three months (66% for BCI; 65% for BBCET) than in the last three months of TB therapy (36% for BCI; 49% for BBCET).

During the course of the study, several unexpected lessons enriched our learning experience. One unexpected benefit of NTX was its use as a “bridge to surgery.” Surgery is a mainstay adjunctive treatment for TB, particularly for individuals with focal, resectable disease. Many individuals with indications for surgery in Russia are unable to undergo surgery due to the excessive risk associated with ongoing heavy drinking, including the inability to follow-up with pre- and postoperative assessments and the morbidity associated with severe postsurgical withdrawal. TB physicians worked closely with patients and surgeons to decrease alcohol intake prior to surgery and follow protocols on perioperative NTX management. Several patients who might otherwise not have been able to tolerate surgery underwent successful procedures, and preparation for surgery provided additional motivation to cut down on drinking.

Another aspect of care that heavily influenced the delivery of alcohol treatment was the treatment site. Alcohol interventions were more easily integrated into inpatient TB care than into outpatient services. Hospital physicians and patients had more time to coordinate care and deliver unhurried behavioral interventions. Patients lived in a “community” while in the hospital, in which common opinion (e.g., favorable attitudes toward the study) enhanced their willingness to participate in study activities. By contrast, challenges to implementation in the ambulatory setting included lower adherence to TB care (including medications and appointments) and less interest on the part of both providers and patients, despite efforts to carry out similar programmatic accompaniment in that setting. Based on discussions with providers, an ideal programmatic strategy for integrating alcohol and TB care would be to target in-patients, particularly those who are likely to stay longer in the hospital due to unstable social situations or multidrug-resistant TB. Furthermore, the goal of the intervention may depend on the setting. For instance, even short-term reduction in alcohol use during the intensive phase of TB treatment could improve the chances of TB cure, whereas achieving long-term remission from alcohol use could require the incorporation of additional strategies, such as training in relapse-prevention skills and facilitating family or community supports for a sober lifestyle.

### Ongoing Challenges

Transitioning from an effectiveness trial to a sustainable programmatic intervention would present additional challenges. If proven effective, sustained integration of alcohol care into TB services would require secure funding for NTX, training, and oversight, as well as an institutional commitment to maintain such interventions as the ongoing standard of care. Moreover, the external validity of any clinical trial—even an effectiveness trial—is limited by inherent differences in participant characteristics, including their motivation to participate, the influence of study compensation, and any indirect support received due to participation in the study. Finally, Tomsk Oblast Tuberculosis Services has served as a model of innovative programs for the past two decades, including early adoption of DOTS and the implementation of a model program for multidrug-resistant TB. Adapting the study interventions to other TB programs in Russia and elsewhere requires a package of “deliverables” designed for dissemination, such as materials for training and patient care, mechanisms for monitoring and evaluation, and financial resources.

## DISCUSSION

The IMPACT study involved the implementation of two evidence-based alcohol interventions in a novel context: integrated into TB services in a resource-limited setting. The process of intervention development, training, and delivery highlights key considerations for global efforts to apply evidence-based interventions to new populations and health infrastructures, particularly when adapting an EBI that has been well studied in upper-income countries but not in resource-limited settings.

The primary objective of adapting an EBI to a different cultural context is to produce the cultural equivalent of a model intervention program. Research is mixed but has demonstrated the benefit of adapting EBIs, rather than using the original EBI^45^ or creating new programs de novo.^46^ The EBI can be modified in form (characteristics of delivery person, channel, or location of delivery)^47^ or in content.^48^ Content can itself be modified superficially or on a deeper, structural level.^49^ Several models for EBI adaptation have been developed, such as the Diffusion of Effective Behavioral Interventions (DEBI) project by the Centers for Disease Prevention and Control^50^ and the ADAPT-ITT model by the Emory Center for AIDS Research. Both models include needs assessment, selection of preferred EBI, identification of qualified experts, pilot research, and efficacy evaluation.^51^,^52^ Although our study did not explicitly follow either the DEBI or ADAPT-ITT model, we included their common core principles in our process.

Adaptation of EBIs in resource-poor settings presents distinct challenges. Models for adapting EBIs have been generally geared toward cultural minority groups in the United States, but little of the literature addresses the unique challenges of adapting EBIs where resources are scarce.^47^,^53^,^54^ If an intervention is perceived, for example, as irrelevant or culturally inappropriate, failure of local communities to endorse the intervention may result in its failure to prove effective.^45^ This phenomenon may be complicated further in communities that have, in the past, experienced what proved to be failed attempts to implement poorly adapted interventions. Perhaps most importantly, the influence of structural factors—for example, the lack of health services; barriers to accessing health care such as cost and distance; and socioeconomic inequalities leading to greater health risk and less health-seeking behavior among women, the poor, and stigmatized populations—on individual behavior is greatest in resource-limited settings.^55^,^56^ Therefore, strategies to implement evidence-based care in such settings must include not only the intervention but programmatic buttressing to allow vulnerable populations to access and adhere to these services.

In summary, a wealth of literature establishes the efficacy of numerous alcohol interventions. Nonetheless, getting these interventions to work in the “real world” can be challenging—and even more so in the context of implementation in resource-limited settings where socioeconomic inequalities, limited access to health services, and inadequate health infrastructure make treatment of chronic mental health problems difficult. One of the greatest challenges in such settings is delivering longitudinal alcohol care to marginalized individuals who are unlikely to seek help and to remain in treatment for alcohol problems. Nonetheless, our experience implementing alcohol care for TB patients in Tomsk highlights some of the key aspects to effectively disseminating evidence-based care: (1) knowledge of local needs and resources, (2) collaborative effort with iterative local input, (3) delivery of alcohol services as an integral component of treatment for other chronic medical conditions, (4) task shifting to nonspecialized providers who deliver “front-line primary care services” to the community, (5) programmatic “accompaniment” throughout the process of implementation to ensure seamless integration and clinical proficiency, and (6) commitment of programmatic leadership to dedicate financial and human resources to sustained operations.
